# Structured management strategy based on the Gastro-oesophageal Reflux Disease (GERD) Questionnaire (GerdQ) vs. usual primary care for GERD: pooled analysis of five cluster-randomised European studies

**DOI:** 10.1111/j.1742-1241.2012.02992.x

**Published:** 2012-07-16

**Authors:** J Ponce, V Garrigues, L Agréus, E Tabaglio, M Gschwantler, E Guallar, M Tafalla, J Nuevo, J Hatlebakk

**Affiliations:** 1 Digestive Functional Disorders Unit, Hospital Universitario La FeValencia, Spain; 2 Center for Family and Community Medicine, Karolinska InstutitetStockholm, Sweden; 3 Società Italiana Medicina GeneraleBrescia, Italy; 4 WilhelminenspitalVienna, Austria; 5 Department of Epidemiology, Johns Hopkins University Bloomberg School of Public HealthBaltimore, MD, USA; 6 Medical DepartmentAstraZeneca, Madrid, Spain; 7 Haukeland University HospitalBergen, Norway

## Abstract

**Background:**

Response to treatment among primary care patients with gastro-oesophageal disease (GERD) is variable.

**Aim:**

The GERD Management Project (GMP) evaluated the effectiveness of a structured management approach to GERD vs. standard treatment (usual care).

**Methods:**

Data from five cluster-randomised clinical trials in adult primary care patients with symptoms of GERD were pooled. The structured pathway was based on the self-administered GERD Questionnaire (GerdQ) and was compared with standard treatment.

**Results:**

1734 patients were enrolled (structured treatment, *n* = 834; standard treatment, *n* = 900). The difference in the mean GerdQ score change from baseline favoured the structured pathway (−0.61; 95% CI: −0.88, −0.34; p < 0.001). The odds ratio for an indication for treatment revision at the end of follow-up (structured vs. standard treatment) was 0.39 (95% CI: 0.29, 0.52; p = 0.001).

**Conclusions:**

Management of primary care patients with GERD can be improved by systematic stratification of patients using a patient management tool such as the GerdQ.

**What's known**

- The treatment response among primary care patients with gastro-oesophageal reflux disease (GERD) is variable and many patients who receive medical attention for GERD still continue to experience persistent symptoms.
- An unmet need therefore exists for improved management of GERD in primary care, including a means to identify patients who may benefit from further assessment and more effective therapies.

**What's new**

- The GERD Management Project evaluated the effectiveness of a structured management approach to GERD, based on the self-administered GERD Questionnaire (GerdQ) vs. standard treatment (usual care). Data were pooled from five cluster-randomised European clinical trials in 1734 adult primary care patients with symptoms of GERD.
- Findings show that management of primary care patients with GERD can be improved by systematic stratification of patients using a patient management tool such as the GerdQ.

## Introduction

Gastro-oesophageal reflux disease (GERD) is defined by troublesome symptoms and/or complications resulting from the reflux of stomach contents into the oesophagus (1). It occurs relatively commonly, with a prevalence of 10–20% in the general population in the western world (2,3). GERD is associated with a substantial impairment of patients’ daily lives (4), health-related quality of life (5) and work productivity including absenteeism and decreased productivity while working (5,6). Indeed, a recent report showed that GERD was associated with mean monetary losses of €55–273 per patient per week related to work absenteeism and reduced productivity across a number of European countries (6). Furthermore, patients with GERD have increased use of prescription and over-the-counter (OTC) medications (5,7), increased consultation rates (8) and increased use of healthcare resources and total healthcare costs (5).

The treatment response among primary care patients with GERD is variable, and many patients who receive medical attention for GERD still continue to experience persistent symptoms (7,9,10) with associated substantial impairment of their daily lives (4,9). This variable treatment response may relate to a number of factors, including the accuracy of primary care diagnosis; tailoring of treatment to the patient’s symptom severity; the presence of a suitable follow-up to assess treatment success; and national treatment guidelines, restrictions and traditions. Recent data, however, indicate that the management of patients with GERD in patients in Europe is suboptimal (11). While several clinical practice guidelines have been developed to this end by different medical societies and consensus groups (12–17), their impact on physician behaviour has generally been limited (18–22).

It is clear that there is an unmet need for improved management of GERD in primary care, including a means to identify patients who may benefit from further assessment and more effective therapies. The GERD Management Project (GMP) was designed to evaluate the effectiveness of a structured management approach to GERD compared with standard treatment (usual care), pooling individual patient data from five related studies conducted in Europe (23).

## Methods

### Study design

**Figure 1:**
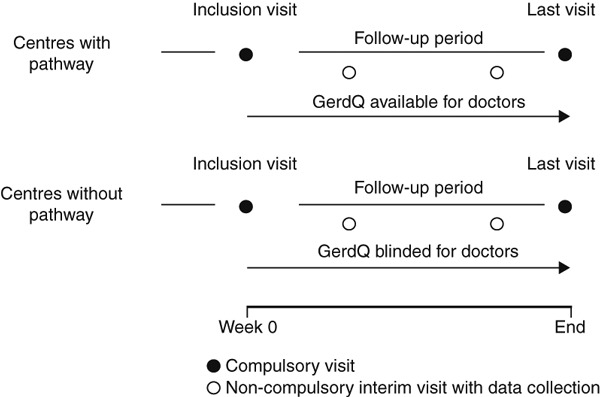
Study Design. GerdQ, Gastro-oesophageal Reflux Disease Questionnaire. Adapted from Ponce et al. (23); copyright © 2011, reprinted by permission of SAGE

Individual patient data from five cluster-randomised clinical trials (ClinicalTrials.gov NCT00842387) conducted during 2009 at multiple study centres in Austria, Italy, Norway, Spain and Sweden were pooled to compare the efficacy of a new structured treatment pathway for primary care patients with GERD with existing treatment practice for improving clinical outcomes. The Methods of this study have been previously reported in detail (23). Briefly, adult (aged ≥ 18 years) male and female primary care patients with symptoms suggestive of GERD, irrespective of severity, were recruited into the studies. Patients who had alarm symptoms were not eligible for inclusion. All trials used a standard study protocol (Figure 1) based on the GERD Questionnaire (GerdQ) with regional modifications to meet regional guidelines and regulatory requirements (Table 1).

**Table 1:** Key regional standard protocol variations. Adapted from Ponce et al. [23]; copyright © 2011, reprinted by permission of SAGE

Country	Protocol variation	Reason for variation
Austria	Second (follow-up) visit conducted 2–3 weeks after baseline, rather than 4 weeks	National guidelines for empirical treatment of GERD and local input from specialists
Spain	RDQ used to evaluate primary study objective, not EQ-5D or WPAI-GERD	Local preference for RDQ
Italy	Study classed as interventional due to protocol requirement to administer esomeprazole to patients identified as having ‘high impact’ GERD	Regulatory requirement National guidelines on GERD management exist, but their implementation is not mandatory
Norway	Gastroenterology specialists selected as investigators, rather than primary care physicians who must refer GERD patients	National GERD guidelines make endoscopic examination (±pH-metry) mandatory for reimbursement of treatment costs
Sweden	Extended follow-up of 5 months ± 4 weeks RDQ used to evaluate primary study objective, in addition to the EQ-5D and WPAI-GERD Minor variations in treatment options	Request to measure HRQL and work productivity, before and after treatment Local preference for RDQ GERD guidelines vary on a county-by-county basis

EQ-5D, EuroQoL-5D Questionnaire; GERD, gastro-oesophageal reflux disease; HRQL, health-related quality of life; RDQ, Reflux Disease Questionnaire; WPAI-GERD, Work Productivity and Activity Impairment Questionnaire for patients with GERD.

The GerdQ is a self-administered, patient-centred tool designed for use by healthcare professionals to not only diagnose but also to manage patients with GERD. Indeed, the GerdQ is useful in guiding treatment decisions by differentiating GERD patients with occasional reflux symptoms from patients with frequent symptoms (24) and in monitoring the effect of treatment on patients’ symptoms and daily lives. In brief, the GerdQ scores the frequency of six items (heartburn, regurgitation, dyspepsia, nausea, need for OTC treatment and sleep disturbance) during the past 7 days according to a 4-point scale ranging from 0 to 3 (where 0 = 0 days/week and 3 = 4–7 days/week). Of the six GerdQ items, two items measure the impact of symptoms on patients’ daily lives (need for OTC treatment and sleep disturbance). The remaining four items (heartburn, regurgitation, need for OTC treatment and sleep disturbance) are used to monitor and evaluate treatment response. A score of 2 or 3 in any of these items is an indication for treatment revision.

The structured treatment pathway tested in the GMP trials was based on the GerdQ to identify patients with a high probability of having GERD (GerdQ score ≥ 8). In the structured pathway group, patients with a GerdQ impact score ≥ 3 (≥ 4 in Norway) were classified as high impact GERD group and treated with esomeprazole 40 mg once daily, whereas patients with a GerdQ impact score ≤ 2 (≤ 3 in Norway) were classified as low/moderate impact GERD group and treated with generic proton pump inhibitors (PPIs) according to local guidance. Patients in the standard treatment group (control) received usual care.

### Study outcomes

Efficacy data were collected after 4 weeks of follow-up. Treatment response was determined by the overall score of the four items of the GerdQ used for treatment monitoring (heartburn, regurgitation, need for OTC treatment and sleep disturbance) and by evaluating the proportion of patients with an indication for treatment revision at the end of follow-up (defined as patients scoring 2 or 3 for the items of heartburn, regurgitation, need for OTC treatment or sleep disturbance, that is, on more than 1 day during the previous 7 days). In addition, the changes in each individual GerdQ item used for monitoring treatment were analysed as secondary endpoints.

### Statistical analyses

Differences in baseline characteristics of study patients by treatment group were calculated using *t*-tests or χ^2^ tests, as appropriate. The efficacy of the structured treatment pathway, in relation to usual care (control), was evaluated in terms of the change in GerdQ score between baseline and follow-up and the proportion of participants with an indication for treatment revision at the follow-up visit. The change in GerdQ score (follow-up – baseline) comparing the two groups was estimated using mixed linear models with random intercepts to take clustering by study centre (clinic) into account. All analyses were adjusted by country and by baseline GerdQ scores. In addition, we estimated the treatment effect in a model further adjusted by age (continuous), sex, smoking (current vs. non-current) and alcohol intake (current vs. non-current). Similar analyses were conducted for each of the four individual items of the GerdQ score used for treatment monitoring.

To compare the proportion of participants with an indication for treatment revision at the follow-up visit in the two groups, we used mixed logistic models with random intercepts to take clustering by study centre (clinic) into account. Odds ratios for recommendation of treatment revision at follow-up comparing the implementation vs. control groups were estimated in models adjusted for centre and baseline GerdQ score, and then in models further adjusted by age (continuous), sex, smoking (current vs. non-current) and alcohol intake (current vs. non-current).

Subgroup effects by country, age (< 60 and ≥ 60 years), sex, smoking (current and non-current) and alcohol intake (current and non-current) were estimated by introducing product terms of study variables in fully adjusted mixed linear or logistic models, as appropriate. p-values for the interactions were obtained by testing for the statistical significance of these product terms. All p-values reported were two-sided. Results were considered statistically significant if the two-sided p-value was < 0.05. Statistical analyses were conducted using stata, version 11 (StataCorp, College Station, TX, USA).

## Results

### Patients

**Figure 2:**
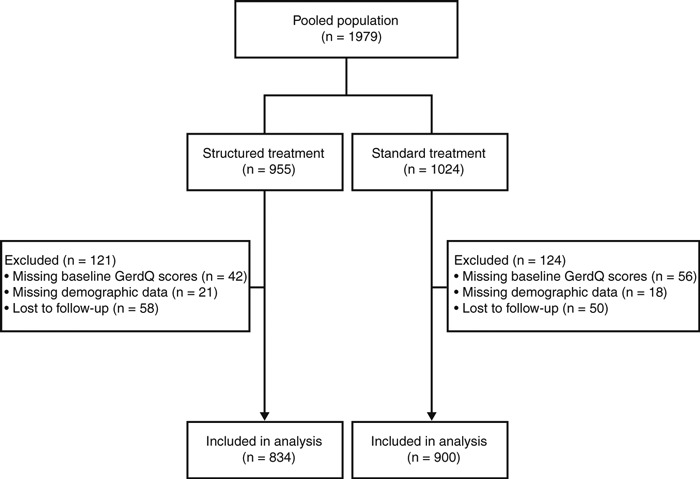
Patients eligible for analysis. GerdQ, Gastro-oesophageal Reflux Disease Questionnaire

As shown in Figure 2, the five trials pooled in this analysis included a total of 1979 patients: 1024 in the standard treatment (control) group and 955 patients in the structured pathway (implementation) group. The final number of patients included in the analysis was 1734: 900 were patients recruited across 115 centres in the standard treatment group and 834 patients recruited across 131 centres in the structured pathway group.

On average, patients in the standard treatment group were slightly younger and more likely to be current drinkers compared with patients in the structured pathway group. There were no significant differences between the two groups in the overall distribution by sex, smoking or date of diagnosis (Table 2). Detailed patient characteristics by treatment group and country are shown in Appendix Table 1.

**Table 2:** Characteristics of study participants by treatment group

	Treatment group*		
	
	Control (*n* = 900)	Implementation (*n* = 834)	p-value
Country			
Austria	308 (34.2)	277 (33.2)	–
Italy	171 (19.0)	164 (19.7)	
Norway	165 (18.3)	170 (20.4)	
Spain	128 (14.2)	153 (18.4)	
Sweden	128 (14.2)	70 (8.4)	
Number of centres	115	131	–
Age, years	52.6 (15.1)	54.2 (15.4)	0.03
Sex, male	427 (47.4)	386 (46.3)	0.63
Current smokers	235 (26.1)	233 (27.9)	0.39
Current drinkers	343 (38.1)	276 (33.1)	0.03
Date of diagnosis†			
Before 2009	349 (55.5)	281 (53.6)	0.49
2009	277 (44.3)	243 (46.4)	

Values are number of patients (%), except for age [mean (SD)].

* Control = usual care; implementation = structured treatment pathway.

† Based on *n* = 1149 patients with available date of diagnosis. 2009 was the cut-off as this coincided with when the studies were conducted.

### GerdQ results

At baseline, the overall average (SD) GerdQ scores using only the four positive items in the control and structured pathway groups were 6.23 (2.94) and 6.68 (2.67), respectively (p < 0.001) (Table 3). At the follow-up visit, the changes (SD) in GerdQ scores (follow-up – baseline) were −3.63 (3.30) and −4.90 (3.17), respectively (p < 0.001). Changes for individual components of the GerdQ score are shown in Appendix Table 2. In mixed linear models with random intercepts for study centre, adjusted by country and baseline GerdQ score, the efficacy of structured pathway vs. control in GerdQ score change was −0.61 (95% confidence interval −0.88 to −0.33; p < 0.001) (Table 4). Further adjustment for age, sex, smoking and alcohol intake did not materially alter the estimates (efficacy −0.61; 95% confidence interval −0.88 to −0.34; p < 0.001).

**Table 3:** GerdQ scores by country and treatment group

	Treatment group*	

	Control	Implementation
Austria		
Number of patients	308	277
Baseline	6.83 (2.77)	7.44 (2.68)
Follow-up	2.56 (2.30)	2.12 (1.84)
Change (follow-up – baseline)	−4.28 (2.96)	−5.32 (3.19)
Italy		
Number of patients	171	164
Baseline	6.36 (3.02)	5.70 (2.49)
Follow-up	2.94 (2.82)	1.51 (1.93)
Change (follow-up – baseline)	−3.42 (3.44)	−4.19 (2.98)
Norway		
Number of patients	165	170
Baseline	5.91 (2.86)	6.21 (2.64)
Follow-up	1.81 (2.35)	1.50 (2.09)
Change (follow-up – baseline)	−4.10 (3.20)	−4.71 (3.24)
Spain		
Number of patients	128	153
Baseline	6.09 (2.94)	6.88 (2.51)
Follow-up	2.63 (2.47)	1.76 (2.39)
Change (follow-up – baseline)	−3.47 (3.33)	−5.12 (3.13)
Sweden		
Number of patients	128	70
Baseline	5.16 (2.98)	6.73 (2.55)
Follow-up	3.27 (2.87)	1.89 (2.37)
Change (follow-up – baseline)	−1.89 (3.33)	−4.84 (3.26)
Overall		
Number of patients	900	834
Baseline	6.23 (2.94)	6.68 (2.67)
Follow-up	2.60 (2.56)	1.79 (2.08)
Change (follow-up – baseline)	−3.63 (3.30)	−4.90 (3.17)

Values are means (SD).

* Control = usual care; implementation = structured treatment pathway. GerdQ, Gastro-oesophageal Reflux Disease Questionnaire.

**Table 4:** Efficacy of implementation (structured treatment pathway) vs. control (usual care) in changing GerdQ scores

	Average difference in GerdQ score change (Implementation – Control)			

	*n*	Efficacy	(95% CI)	p-value
Overall GerdQ*	1734	−0.61	(−0.88 to −0.33)	< 0.001
Overall GerdQ	1734	−0.61	(−0.88 to −0.34)	< 0.001
Heartburn	1734	−0.20	(−0.30 to −0.11)	< 0.001
Regurgitation	1734	−0.20	(−0.29 to −0.11)	< 0.001
Difficulty in sleep	1734	−0.13	(−0.21 to −0.06)	0.001
Additional OTC medication	1734	−0.11	(−0.19 to −0.03)	0.008

Results in the table were estimated from mixed linear models with random intercepts for study centre (clinic). A negative estimate indicates that the implementation group was superior to the control group in reducing GerdQ scores.

* Adjusted by country and baseline GerdQ score. All other analyses were further adjusted for age, sex, smoking and alcohol intake. CI, confidence interval; GerdQ, Gastro-oesophageal Reflux Disease Questionnaire; OTC, over the counter.

**Figure 3:**
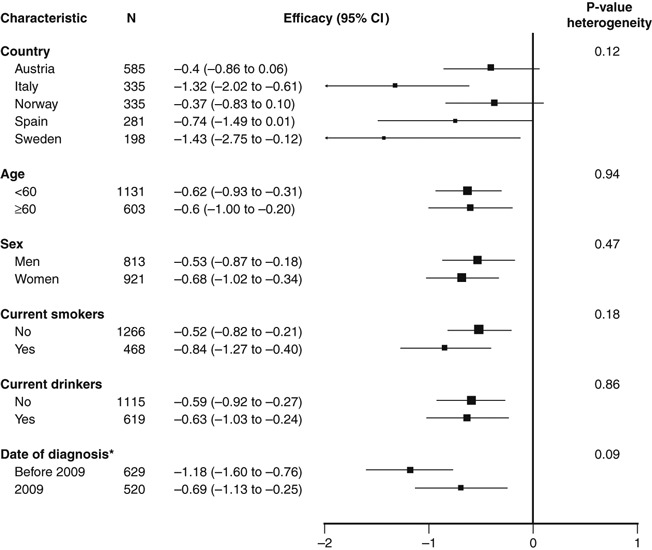
Efficacy of implementation (structured treatment pathway) vs. control (usual care) in improving GerdQ scores. Results were estimated from mixed linear models with random intercepts for study centre. A negative estimate indicates that the structured treatment pathway was superior to usual care in reducing GerdQ score. *Based on 1149 patients with available date of diagnosis. CI, confidence interval

Individual GerdQ items also decreased to a greater extent in the structured pathway group compared with standard treatment (Table 4). Efficacy estimates for individual items were −0.20 (95% confidence interval −0.30 to −0.11) for heartburn, −0.20 (−0.29 to −0.11) for regurgitation, −0.13 (−0.21 to −0.06) for difficulty in sleep and −0.11 (−0.19 to −0.03) for additional OTC medications. The efficacy of the structured treatment pathway was similar across countries and across subgroups defined by age, sex, smoking, alcohol intake and date of diagnosis, with no significant interactions (Figure 3).

**Figure 4:**
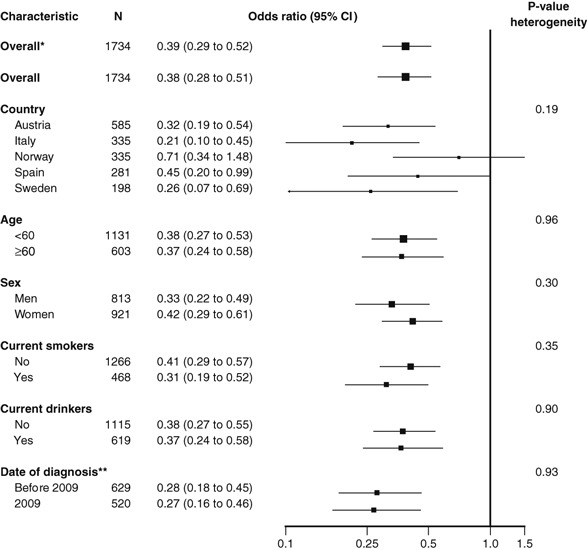
Odds ratios for an indication for treatment revision at the end of follow-up. Results were estimated from mixed logistic models with random intercepts for study centre. An odds ratio < 1 indicates that structured treatment pathway (implementation) group had a lower proportion of participants with an indication for treatment revision at the follow-up visit vs. usual care (control) group. *Stratified by country and adjusted for baseline GerdQ score. All other analyses were further adjusted for age, sex, smoking and alcohol intake. **Based on 1149 patients with available date of diagnosis. CI, confidence interval

At baseline, the overall proportions of participants with indication for treatment revision in the control and structured pathway groups were 88.1% and 93.7%, respectively (p < 0.001) (Table 5). The corresponding proportions at the follow-up visit were 40.2% and 19.9%, respectively (p < 0.001). In mixed logistic models with random intercepts for study centre, adjusted by country and baseline GerdQ score, the odds ratio for treatment revision at the follow-up visit comparing the structured pathway and control groups was 0.39 (95% confidence interval 0.29 to 0.52; p = 0.001) (Figure 4). Further adjustment for age, sex, smoking, alcohol intake and date of diagnosis did not modify the results. The odds ratios were also similar across countries and across subgroups defined by age, sex, smoking, alcohol intake and date of diagnosis with no significant interactions (Figure 4).

**Table 5:** Number (proportion) of participants with indication for treatment revision by country and treatment group

	Treatment group*	
	
	Control	Implementation
Austria		
Total number of patients	308	277
*N* (%) with indication for revision		
Baseline	293 (95.1)	264 (95.3)
Follow-up	111 (36.0)	46 (16.6)
Italy		
Total number of patients	171	164
*N* (%) with indication for revision		
Baseline	149 (87.1)	148 (90.2)
Follow-up	81 (47.4)	29 (17.7)
Norway		
Total number of patients	165	170
*N* (%) with indication for revision		
Baseline	147 (89.1)	154 (90.6)
Follow-up	53 (32.1)	39 (22.9)
Spain		
Total number of patients	128	153
*N* (%) with indication for revision		
Baseline	105 (82.0)	146 (95.4)
Follow-up	48 (37.5)	33 (21.6)
Sweden		
Total number of patients	128	70
*N* (%) with indication for revision		
Baseline	99 (77.3)	69 (98.6)
Follow-up	69 (53.9)	19 (27.1)
Overall		
Total number of patients	900	834
*N* (%) with indication for revision		
Baseline	793 (88.1)	781 (93.7)
Follow-up	362 (40.2)	166 (19.9)

* Control = usual care; implementation = structured treatment pathway.

## Discussion

The results of this large-scale pooling project of five cluster-randomised trials showed that stratification of patients according to GerdQ scores, using a locally adapted primary care management strategy, significantly increased the likelihood of a response to treatment compared with usual clinical practice. Indeed, patients who underwent treatment through the structured treatment pathway had significantly greater improvements (reductions) in GerdQ scores at follow-up than patients treated via the standard pathway. Also, patients in the structured treatment pathway were significantly less likely to have an indication for treatment revision at follow-up than those who received usual care. These results were robust and were not affected by adjustments for patient baseline factors including country of origin and baseline GerdQ score.

Symptomatic GERD is associated with substantial impairment of patients’ daily lives (4), health-related quality of life (5), work productivity and costs (5,6). As many patients with GERD who undergo primary care treatment often continue to experience persistent symptoms (7,9,10), it is evident that the management of patients with GERD in primary care is suboptimal. The positive findings observed in the structured management group compared with usual care, including improvements in GerdQ score and decreased need for treatment revisions, are likely due to an improved ability by physicians to quantify the impact of GERD and to tailor the treatment plan accordingly to the needs of each individual patient.

The GerdQ consists of items from the Reflux Disease Questionnaire, the Gastrointestinal Symptom Rating Scale and the GERD Impact Scale, all of which have been previously validated and shown to have high accuracy in GERD (25–27). The high accuracy, together with its brevity and ease of use, makes the GerdQ an ideal tool for the management of patients with GERD in primary care. It is important to emphasise, however, that GerdQ is a relatively simple management tool, and therefore, patients may need to be investigated further in certain circumstances (e.g. failure to respond to therapy, which would be evident in terms of no decrease in GerdQ score). In such cases, specialist referral is warranted for further testing according to locally preferred methods (e.g. manometry, pH-metry). Indeed, a number of country-specific variations were included in the GMP protocol to accommodate local preferences and national patient management guidelines across the five European countries included in this project. In spite of the differences in study protocol and intervention, there were no significant differences in the efficacy of the structured treatment pathway across countries, demonstrating that the underlying treatment protocol can be locally adapted to the clinical and healthcare delivery circumstances of specific countries and still maintain high efficacy. This approach suggests that the structured treatment pathway can be extended to other countries with variable local guidelines and treatment traditions.

The strengths of the GMP lie in its size, its cluster-randomised design and the fact that this project was developed through a unique modifiable approach, allowing local regulations in each country to be met. Indeed, local healthcare authorities and physicians liaised during the development of each country-specific protocol. Some limitations of the study include the impossibility of blinding the study interventions and the lack of information on compliance with the structured treatment pathway and usual care strategies by individual patients. It also has to be considered that the study considered all GERD patients together, whereas there are phenotypes of GERD that may respond differently to acid-suppressive therapy. For example, the majority of patients with reflux oesophagitis respond well to PPI therapy, while the efficacy of such therapy in patients with non-erosive reflux disease and functional heartburn is modest (28–30). The fact that GERD has different phenotypic responses to acid-suppressive therapy should be borne in mind, as the GerdQ evaluation does not take this detail into account. Moreover, the questionnaire does not consider the impact of dysphagia, a relatively common symptom in patients with GERD (31).

In conclusion, management of patients with GERD in primary care can be greatly improved by systematic use of a patient management tool such as the GerdQ. Local adaptations of this primary care management strategy were able to be implemented across a range of European countries with variable preferences and national treatment guidelines, suggesting that this strategy is widely applicable and may result in significant clinical benefit for GERD patients.

## Author contributions

Study concept and design: Ponce, Garrigues, Agréus, Gschwantler, Tafalla, Nuevo and Hatlebakk. Data collection: Ponce, Garrigues, Agréus, Tabaglio, Gschwantler and Hatlebakk. Design of the pooled analyses and statistical analysis: Guallar. All authors contributed to data interpretation and drafting/critical revision of the manuscript for important intellectual content and approved the final version for submission.

## Funding and acknowledgements

This study was funded by AstraZeneca, Madrid, Spain. We thank Anna Mett of inScience Communications, Springer Healthcare, who provided medical writing support funded by AstraZeneca.

## Appendix

**Table 1:** Characteristics of study participants by country and treatment group

	Austria			Italy			Norway			Spain			Sweden		
					
	C	I	p-value	C	I	p-value	C	I	p-value	C	I	p-value	C	I	p-value
Number of patients	308	277		171	164		165	170		128	153		128	70	
Number of centres	60	68		19	18		17	18		16	24		3	3	
Age, years	55.5	57.8	0.07	50.8	52.9	0.19	47.5	50.1	0.12	51.0	50.2	0.64	56.4	61.9	0.009
	(15.6)	(14.9)		(14.4)	(14.2)		(14.7)	(15.3)		(13.6)	(15.0)		(14.0)	(14.6)	
Sex, male	164	136	0.32	71	73	0.58	90	86	0.47	46	69	0.12	56	22	0.09
	(53.3)	(49.1)		(41.5)	(44.5)		(54.6)	(50.6)		(35.9)	(45.1)		(43.8)	(31.4)	
Current smokers	117	104	0.91	36	32	0.73	37	46	0.33	27	42	0.22	18	9	0.81
	(38.0)	(37.6)		(21.1)	(19.5)		(22.4)	(27.1)		(21.1)	(27.5)		(14.1)	(12.9)	
Current drinkers	57	38	0.12	60	62	0.61	121	120	0.58	4	10	0.19	101	46	0.04
	(18.5)	(13.7)		(35.1)	(37.8)		(73.3)	(70.6)		(3.1)	(6.5)		(78.9)	(65.7)	
Diagnosis in 2009*	18	25	0.04	64	66	0.60	108	55	0.05	85	95	0.45	2	2	0.11
	(11.3)	(20.3)		(37.4)	(40.2)		(85.7)	(74.3)		(66.4)	(62.1)		(4.9)	(20.0)	

Values are number of patients (%), except for age [mean (SD)]. C, control (usual care); I, Implementation (structured treatment pathway).

* Date of diagnosis was available in the following number of patients: Austria: 159 Control, 123 Implementation; Italy: 171 Control, 164 Implementation; Norway: 126 Control, 74 Implementation; Spain: 128 Control, 153 Implementation; Sweden: 41 Control, 10 Implementation.

**Table 2:** Individual components of GerdQ scores at baseline and at the follow-up visit

	Treatment group*	
	
	Control	Implementation
Heartburn		
Number of patients	900	834
Baseline	2.08 (1.00)	2.21 (0.86)
Follow-up	0.92 (0.93)	0.67 (0.79)
Change (follow-up – baseline)	−1.16 (1.22)	−1.54 (1.10)
Regurgitation		
Number of patients	900	834
Baseline	1.64 (1.06)	1.63 (0.99)
Follow-up	0.75 (0.87)	0.49 (0.70)
Change (follow-up – baseline)	−0.89 (1.10)	−1.14 (1.07)
Difficulty in sleep		
Number of patients	900	834
Baseline	1.40 (1.06)	1.49 (1.03)
Follow-up	0.52 (0.80)	0.34 (0.61)
Change (follow-up – baseline)	−0.89 (1.12)	−1.15 (1.07)
Additional OTC medication		
Number of patients	900	834
Baseline	1.11 (1.20)	1.36 (1.19)
Follow-up	0.42 (0.83)	0.29 (0.66)
Change (follow-up – baseline)	−0.69 (1.27)	−1.07 (1.26)

Values are means (SD).

* Control = usual care; implementation = structured treatment pathway. GerdQ, Gastro-oesophageal Reflux Disease Questionnaire; OTC, over the counter.
